# Recent Developments in Deep Eutectic Solvents Applications in Liquid Chromatography: 2019–2025

**DOI:** 10.1002/jssc.70160

**Published:** 2025-05-07

**Authors:** Derya Demir, Joanna Antos, František Švec, Hana Sklenářová

**Affiliations:** ^1^ Department of Analytical Chemistry Faculty of Pharmacy in Hradec Králové Charles University Hradec Králové Czech Republic; ^2^ Department of Water Supply and Bioeconomy Faculty of Environmental Engineering and Energy Poznan University of Technology Poznan Poland

**Keywords:** deep eutectic solvents, green chemistry, liquid chromatography, mobile phase additive, separation efficiency, stationary phase modification

## Abstract

Deep eutectic solvents (DES) are used as mobile phase and stationary phase modifiers, or as for the stationary phase itself for thin layer/liquid/supercritical chromatography. Their specific properties can improve the separation selectivity, reduce peak tailing, and shorten the separation time. In terms of environmental impact, the advantages of DES are based on their biodegradability, recyclability, and stability in terms of mechanical/chemical/thermal properties. The disadvantages are related to higher viscosity and degradation in aqueous solutions. This review focuses on works that have been published since 2019, the year the excellent comprehensive review by Cai and Qiu was printed. Selected parameters are discussed that should be considered when DES are preferred over commonly used mobile and stationary phases due to green chemistry trends, while taking into account their limitations in modern LC separations. This review also centres on critical aspects of DES applications in the field of liquid and supercritical fluid chromatographic separations.

## 1 Introduction

In general, deep eutectic solvents (DES) are solutions of Lewis or Brønsted acids and bases that form a eutectic mixture. They have a wide range of potential applications, including, for example, organic synthesis, catalytic, electrochemical, and separation processes. They were originally prepared from a variety of carboxylic acids as hydrogen bond donors and choline chloride. DES are easily prepared using cheap and simple procedures [1], while the resulting product has a lower melting point than each of the components contained [2]. The properties of DES are determined by the number of acid groups, the aryl/alkyl substituents, and the composition of the mixture. The positive features are tunable physicochemical properties that allow the properties of the DES to be tailored to the requirements of the respective application.

Their physical properties and phase behavior are close to those of ionic liquids (IL), as described in a recent review by Plotka‐Wasylka [3]. Thus, DES are expected to be an alternative to IL in certain applications, mainly because of their minimized environmental impact compared to standard solvents. In terms of stability, there is always a risk of decomposition of DES in water, which should be considered when using them in aqueous solutions [4].

Atypical DES solvents are based on halogen bonds instead of hydrogen bonds, which again correspond to the lower melting point of such mixtures. These DES can be formed from phenols or polyols to result in hydrophilic solvents and from terpenoids with fatty alcohols to provide hydrophobic solvents. There is no clear classification whether these solvents still belong to the group of DES or whether they are just binary mixtures with special physicochemical properties. The lack of a strict rule to distinguish between these two groups persists [5].

DES has advantages with respect to their environmental impact, including low toxicity, non‐flammability, non‐volatility, mechanical/chemical/thermal stability, and excellent properties in the solubilization process [4]. Low cost and easy recycling, biodegradability, and thus sustainability, should also be emphasized with respect to the green chemistry principles. On the other hand, they also have a higher viscosity compared to standard solvents, which can play an important role in their applications.

**FIGURE 1:**
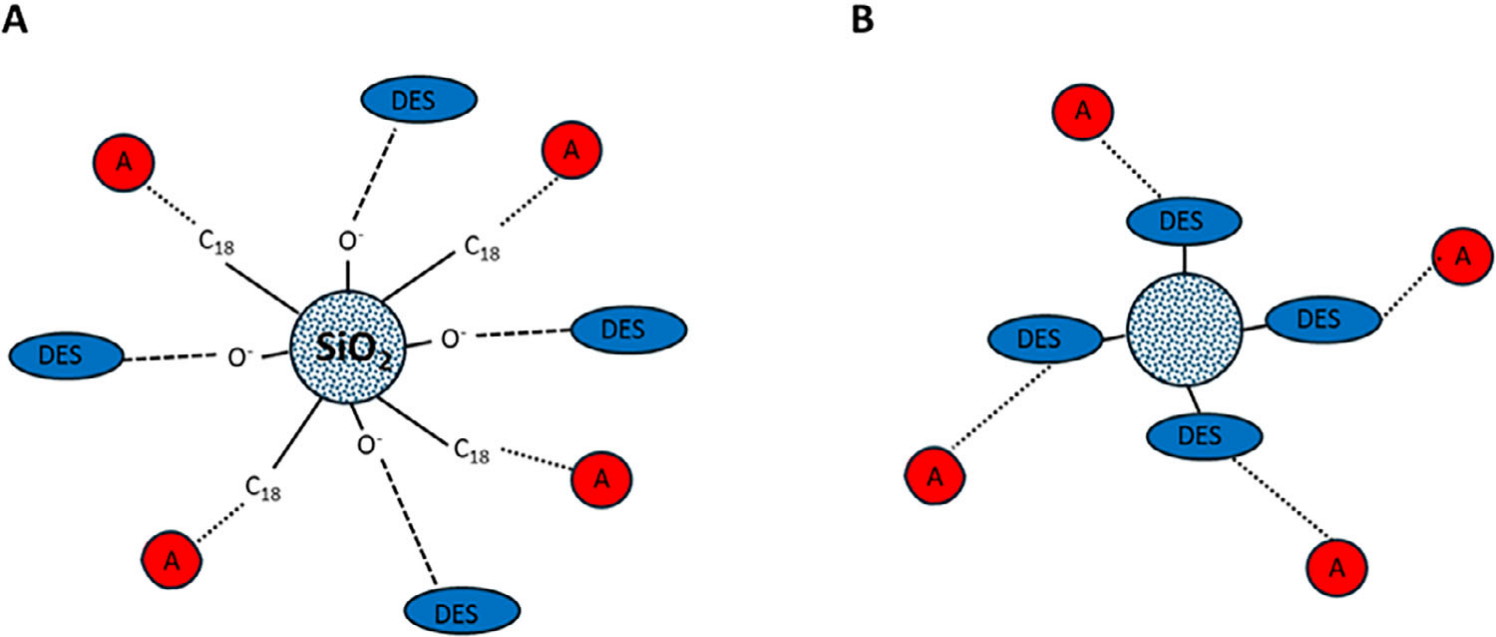
Role of DES as: (A) a mobile phase additive for separation using C18 stationary phase and (B) stationary phase modifier. Letter A in the red circle means analyte.

Focusing on the chromatographic area, DES has been used to facilitate both the separations and the sample preparation via solid‐phase extraction. In both cases, the advantages of DES are noticeable thanks to the improvement in selectivity, increased peak symmetry, and changes in retention [6, 7]. The special properties of DES in chromatographic separations can be explained by the ion‐pairing properties and the ability to interact with residual silanol groups on the surface of silica‐based stationary phases [8]. The principle of DES modification as a mobile phase additive or stationary phase modifier is depicted in Figure 1. DES can also be used to prepare a new stationary phase based on polymer particles with sufficient mechanical stability to improve the separation of structurally similar analytes, including enantiomers [9]. As additives in the mobile phase, they can act as hydrogen acceptors or hydrogen bond donors and, even at low concentrations, improve peak resolution and their symmetry [6].

The above advantages make DES desirable in the field of chromatographic separation as additives to the typical mobile phases to reduce the content of organic solvents or as their complete alternatives in TLC/SFC to increase the greenness and quality of the separation.

## 2 DES as a Mobile Phase Additive

The typical mobile phase in the most commonly used reversed‐phase LC separations consists of an organic solvent, most often acetonitrile (ACN), mixed with an aqueous component. Modern trends in greening LC separations focus on reducing the percentage of organic solvents in the mobile phase, for example, by using pure aqueous phase or higher temperatures. DES as an additive to the aqueous mobile phase is an alternative to such approaches, as it can decrease the amount of organic solvent used [10]. Certain problems are then based on higher viscosity of DES that can be overcome using lower concentrations in mobile phases or tuning this parameter [11].

DES recently used as mobile phase additives are listed in Table 1. These applications demonstrate that DES allowed separation in different chromatographic modes. For example, thin‐layer chromatography (TLC) using a mixture of methanol and DES allowed the separation of alkaloids with a distinct zone sharpening observed by densitometric detection [12, 13]. Blocking of free silanol groups occurred when DES was used as a mobile phase additive during separations in particle‐packed and monolithic reversed‐phase C18 columns. The separations were most positively affected when the analytes of interest were basic in nature, such as alkaloids [14] and other such substances. In general, the tailing of peaks of basic compounds in LC was significantly suppressed, and the retention was shorter. This resulted in higher column efficiency and shorter analysis times.

**TABLE 1:** DES as mobile phase additives in thin‐layer chromatography (TLC), liquid chromatography (LC), and supercritical fluid chromatography (SFC).

Analyte	Stationary phase	Mobile phase	DES	Mode	Time (min)	WAC score	Ref
*Chelidonium maius* alkaloids (5)^a^	Si60 plate	20% MeOH 35% MeOH	Cam:Phe 2:1 Men:Lim 2:1 Men:Phe 1:1	TLC	—	—	[12, 13]
Melamine in cow milk	C18 (150 × 4.6 mm, 5 µm)	SDS (0.10 mol/L), 4% (v/v) DES, 4% (v/v) glacial acetic acid 92:4:4, pH 3	ChCl:EG 1:2	Isocratic MLC	10	92.7	[15]
Aspirin, atorvastatin, metformin, metoprolol	C18 (150 × 4.6 mm, 5 µm)	SDS (0.09 mol/L): BuOH:DES:GAC 83:10:3.5:3.5	ChCl:EG 2:1	Isocratic MLC	12	90.8	[16]
Imidocarb dipropionate residues	C18 monolith (50 × 4.6 mm)	SDS (0.075 mol/l): EtOH:DES (40:50:10, v/v/v), pH 4	ChCl:EG 1:2	Isocratic MLC	1.2	96.0	[17]
Protocatechuic acid and derivatives (9)^a^	C18 monolith (100 × 4.6 mm)	water (A), EtOH (B), and DES (C)	LA:Glu:H_2_O 5:1:4	Gradient LC	35	—	[18]
Biogenic amines in wine (8)^a^	C18 (250 × 4.6 mm, 5 µm)	0.73% DES ‐ 65% acetonitrile	ChCl:EG 1:3	Gradient LC	20	88.5	[19]
Phenolic substances (6)^a^	C18 (250 × 4.6 mm, 5 µm)	0.06 mol/L SDS, 0.01 mol/L Brij‐35, 5% (v/v) DES, pH 2.90	Betaine:EG 1:2.5	Isocratic MLC	15	91.9	[20]
Isoflavons (5)^a^	C18 (250 × 4.6 mm, 5 µm)	Water (A), ACN (B), 0.1%	ChCl:CA 1:1	Gradient LC	50	74.9	[21]
Isoquinoline alkaloids (10)^a^	RX‐SIL (150 × 2.1 mm, 5 µm)	CO_2_, MeOH‐2% H_2_O‐0.5% FA‐0.25% DES	ChCl:Gly 2:1	Gradient SFC	25	91.5	[14]
Model mixtures of pharmaceuticals	Polysaccharide Chiralpak (100 × 4.6 mm, 5 µm)	CO_2_, MeOH‐0.2% DES	ChCl:Glu	Isocratic SFC	—	—	[22]

^a^The number of compounds separated.

Abbreviations: ACN, acetonitrile; CA, citric acid; Cam, camphor; ChCl, choline chloride; EG, ethylene glycol; EtOH, ethanol; FA, formic acid; Glu, glucose; Gly, glycerol; LA, lactic acid; Lim, limonene; Men, menthol; MeOH, methanol; MLC, micellar liquid chromatography; Phe, phenol; WAC, White Analytical Chemistry.

**FIGURE 2:**
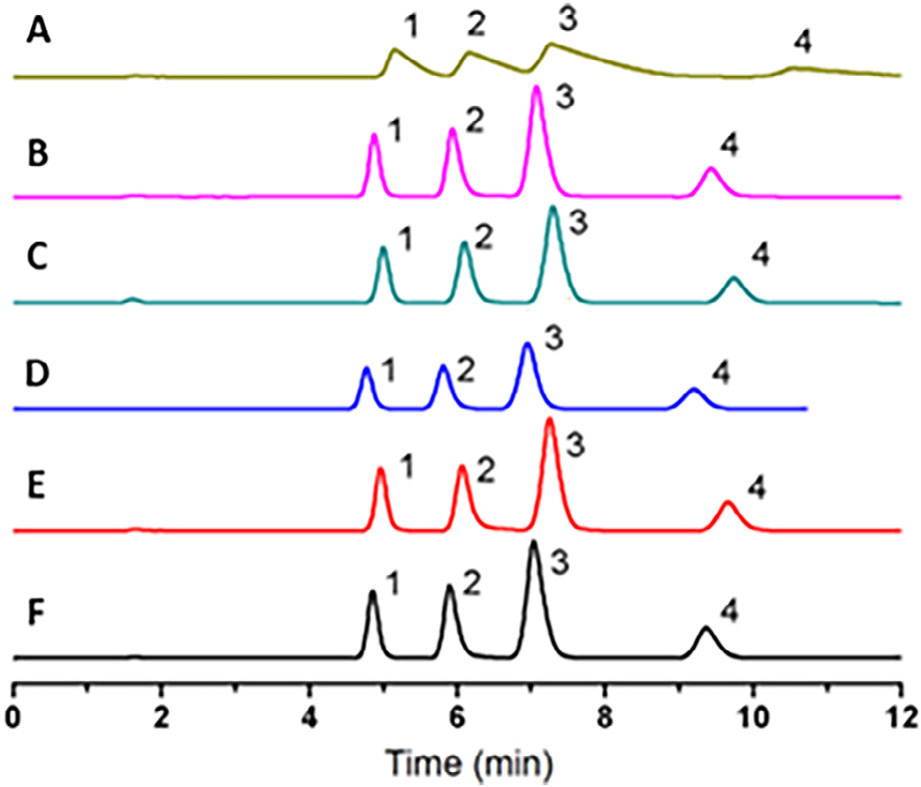
Separation of quaternary alkaloids using different mobile phases. (A) 32:68 (v/v) ACN:DES:0.57% ethylene glycol (pH 3.3), (B) 32:68 (v/v) ACN:DES:0.43% choline chloride (pH 3.3), and 32:68 (v/v) ACN:1.0% DES (pH 3.3) for DES: (C) 1% choline chloride:ethylene glycol 1:3, (D) 1% choline chloride:urea 1:2, (E) 1% choline chloride:citric acid 1:1, (F) 1% choline chloride:glycerol 1:2. Conditions: stationary phase Shimadzu CTO‐15C C18 ODS column (150 × 4.6 mm, 5 µm); UV detection 345 nm; temperature 30°C; injection volume 10 µL; flow rate 1 mL/min. Peaks: 1 coptisine chloride; 2 sanguinarine; 3 berberine chloride; 4 chelerythrine. Reproduced with permission from ref. [6].

However, there were also some problems. For example, Tan et al. investigated the effect of adding DES of different compositions to the mobile phase composed of aqueous ACN. Figure 2 shows similar chromatograms using a mobile phase containing only choline chloride compared to DES formed from choline chloride and ethylene glycol, urea, citric acid, and glycerol, respectively [6]. It appears that choline chloride was the main additive that changed the separation, most likely because DES was susceptible to decomposition in the aqueous medium.

Similarly, Sutton et al. compared natural DES as additives to aqueous‐organic mobile phases while using ACN, methanol, and ethanol [18]. The separation efficiency obtained with ACN and ethanol for the separation of eight derivatives of protocatechuic acid was practically identical to that of the best optimized DES composed of lactic acid, glucose, and water. The difference in retention time between DES and ACN was only 1%–5%. The mobile phase flow rate was 1.5 mL/min, and then the organic solvent consumption was higher in the 30 min analysis, but the optimization could be achieved very easily.

The White Analytical Chemistry (WAC, [23]) score was evaluated only for separation and mobile phase composition; other parameters characterizing the determination of different analytes in different sample matrices were not included to focus only on the main topic of this review. The final scores consisted of a red part corresponding to the applicability, a green part to the greenness, and a blue part to the cost‐effectiveness. When comparing the methods mentioned in Table 1, we found differences in terms of column length and larger diameter, which means longer separation, higher mobile phase consumption, and thus higher analysis costs. In terms of detection, all methods mentioned were based on UV detection with similar sensitivity and cost: applications focused on complex matrices were evaluated with a higher score than just plant extracts or model mixtures. Such a comparison is always influenced by the main objectives and parameters included and can vary significantly when using different features. Our evaluation can be found in the tables in the Supporting Information. The greenness assessment was based on the numbers of Globally Harmonized System of Classification and Labelling of Chemicals (GHS) pictograms and signal words of each reagent that is used for the mobile phase preparation and waste generation per run. The cost of the mobile phases and the time and sample volume required for each run were used to evaluate the cost/time efficiency of the methods. For the objectivity of the comparison, energy consumption, ease of operation, and other requirements were removed from the final calculation of the WAC scores because only the mobile phase preparation was evaluated.

The main difference that is not visualized by the WAC evaluation is based on the optimization step. If we include the tedious and time‐consuming optimization of the mobile phase composition, the separation with ACN as the first‐choice mobile phase will outperform DES in all cases. This fact is demonstrated in Figure 2 and in reference [18], where the separation efficiency of ACN was the same as that of optimized DES. In addition, the safety, health, environment, and HPLC‐EAT scores were evaluated in this work, where a higher score for ACN means a potential risk. However, considering that the consumption of all reagents for DES preparation and mobile phase optimization is again much higher, ACN may excel with easier optimization, but in routine analysis, DES may get a higher WAC score.

Several of the published reports have compared the separations using DES and conventional organic solvents as mobile phases [1, 8, 24]. Interestingly, the direct comparison often did not show any significant effect, since the use of DES, even under optimal conditions, provided separations equivalent to those achieved with mobile phases containing aqueous ACN or methanol. Thus, DES seems to have an environmental advantage by avoiding/minimizing the consumption of standard LC solvents, following the trends of the greening the analytical chemistry. But is this a sufficient argument for using DES? The quality of chromatographic separations depends not only on the mobile phase but also on the stationary phase, the geometric parameters of the column, and on further conditions such as injection volume, temperature, flow rate, and detection. Other options are currently being explored and implemented that also reduce the consumption of organic solvents while using more traditional means. The use of small‐bore and microcolumns, separations at higher temperatures, or tailored stationary phases are good examples of these efforts. Another way for routine laboratories to reduce organic solvent consumption is to recycle the mobile phase, which is simple and can be applied when small sample volumes are injected onto the column. In contrast, the application of DES requires a complete optimization of the separation conditions, mostly applied in combination with old‐fashioned columns. Obviously, the use of DES is not a panacea for reducing the consumption of standard organic solvents.

While similar problems with DES encountered in LC separations, such as decomposition of the aqueous mobile phase and higher viscosity when used undiluted, are also observed in micellar liquid chromatography, the use of DES as a modifier in supercritical fluid chromatography (SFC) is different [14, 22]. Supercritical carbon dioxide of low viscosity does not contain water, thus diminishing the decomposition of DES. The combination of DES with supercritical carbon dioxide can help to tune the non‐aqueous mobile phase for the separation of hydrophilic analytes, which remains challenging.

## 3 Use of DES as Solvents in Preparation of Stationary Phases

Thanks to the properties of DES including environmental friendliness and specific chemistry, these compounds are also used as solvents in the preparation of stationary phases for chromatography. Table 2 shows recent examples of stationary phases prepared in presence of DES as reaction medium.

**TABLE 2:** DES as solvents used in modifications of stationary phases.

DES	Reaction	Stationary Phase	Examples of analytes	Methodology	Ref
ChCl:LA 1:3	Carbon dots grafting	Sil‐DESCD	Hydrocortisone, prednisolone	RPLC	[26]
ChCl:EG 1:3	Carbon dots grafting	Sil‐Glc‐NCD	Ginsenosides, antibiotics	HILIC	[27]
ChCl:EG 1:3	Thiol‐ene click reaction	PVP/PVA@Sil‐UA	Nucleosides, steroid hormones	HILIC, RPLC	[28]
ChCl:LA 1:3	Aminopropyl‐modification	Sil‐P‐DESCDs	Alkylbenzenes, PAHs, sulphonamides, aromatic amines, phenols, flavonoids, nucleoside bases, alkaloids	HILIC	[29]
ChCl:EG 1:3	Thiol‐ene click reaction	Two copolymer grafted silica Sil‐PDM‐PIA	Nucleosides, nucleobases, saccharides, amino acids	HILIC	[30]
ChCl:EG 1:3	Surface radical chain‐transfer reaction in DES	Poly(itaconic acid)‐grafted silica Sil‐PIA	Nucleobases, nucleosides, saccharides, amino acids, ginsenosides	HILIC	[31]
ChCl:EG 1:2	Schiff‐base condensation reaction, Thiol‐ene click reaction	IL‐COF@SiO_2_	Alkylphenols, steroid hormones, bisphenols, nucleosides/bases, sulphonamides	HILIC, RPLC	[32]
ChCl:EG 1:2	Schiff‐base condensation reaction, mercapto‐alkenyl reaction	PAA/COF@SiO_2_	Nucleosides, alkylphenols in environmental water sample	MMC	[33]
ChCl:EG 1:2	Silanization	MIP‐202@SiO₂	Sulphonamides, nucleosides/bases	HILIC, RPLC	[34]
ChCl:EG	In situ polymerization	DES‐ionogel@SiO_2_	Phthalates in tea, PAHs in lake water	HILIC, RPLC, NPLC	[35]

Abbreviations: ChCl, choline chloride; DES‐Ionogel@SiO_2_, deep eutectic solvent‐based ionogel/silica composite; EG, ethylene glycol; HILIC, hydrophilic interaction chromatography; IL‐COF@SiO_2_, ionic liquid modified‐covalent organic framework bonded silica composite; MIP, molecularly imprinted polymer; MMC, mixed mode chromatography; NPLC, normal phase liquid chromatography; PAA, polyacrylic acid; PAHs, polycyclic aromatic hydrocarbons; PIA, poly‐itaconic acid; PVP/PVA@Sil‐UA, polyvinyl pyrrolidone/polyvinyl alcohol composite hydrogel‐functionalized silica stationary phase; Sil‐DESCD, deep eutectic solvents‐based carbon dots bonded silica; Sil‐Glc‐NCD, glucose‐based nitrogen‐doped carbon dots‐bonded silica; Sil‐NCD, N‐doped carbon dots‐bonded silica.

The wide‐ranging review by Farooq et al. covered, among many other applications, the use of DES as porogens for the preparation of organic polymer‐based monolithic columns and hybrid columns with nanomaterials in capillary electrochromatography [25]. In order to achieve optimal pore structure and pore size of these columns prepared using standard polymerization procedures, porogens including toxic organic solvents, such as toluene‐isooctane and toluene‐isooctane‐dimethyl sulfoxide were required. Due to the environmental and safety concerns associated with these hazardous solvents, DES were investigated as a sustainable alternative. DES helped to increase the surface area of the monolithic stationary phases and facilitated the extent of the analyte interactions thereby improving efficiency and selectivity.

**FIGURE 3:**
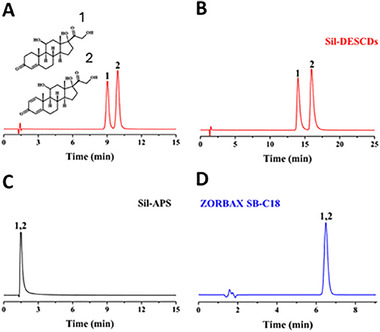
Separations of hydrocortisone (1) and prednisolone (2) using columns packed with silica‐DES carbon dots (DESCD) (A, B), aminopropyl modified silica (Sil‐APS) (C), and ZORBAX SB‐C18 (150 × 4.6 mm, 3 µm) (D) prepared using DES as reaction medium. Conditions: mobile phase (A) acetonitrile–water (7/93, v/v), (B) water, (C) acetonitrile–water (7/93, v/v), (D) acetonitrile–water (25/75, v/v); temperature 30°C; flow rate 1.0 mL/min; detection 254 nm. Reproduced with permission from ref. [26].

In another example, Fu et al. synthesized DES‐based carbon dots (DESCDs) by solvothermal method using choline chloride: lactic acid in 1:3 ratio as carbon source [26]. Then they subsequently chemically modified silica with DESCDs to form a novel hydrophobic chromatographic stationary phase (Sil‐DESCDs) for RPLC. The Sil‐DESCDs column exhibited strong hydrophobic selectivity for analytes such as polycyclic aromatic hydrocarbons (PAHs), alkylbenzenes, aromatic amines, and phenolics. Retention factors for alkylbenzenes decreased using aqueous ACN mobile phases at concentrations increasing from 40% to 60% and with increasing temperatures from 25°C to 45°C, indicating an exothermic retention process. PAHs were best separated using a 28/72 ACN‐water mobile phase. The column also provided baseline separation of structurally similar prednisolone and hydrocortisone using only pure water as the mobile phase as shown in Figure 3.

Although DES were included in the reaction pathway, non‐green chemicals such as toluene, 3‐isocyanatopropyltriethoxysilane, 3‐mercaptopropyltriethoxysilane, and dimethylformamide were used to functionalize the silica‐based stationary phases. Thus, these works represent a certain step towards a greener preparation. However, they do not fully comply with the principles of green chemistry and the greenness of the methods must be questioned.

## 4 DES as a Stationary Phase

There are only a handful of recent papers reporting the use of DES as a part of the stationary phase. The lack of more and deeper studies is probably due to the known instability of DES in aqueous media. In a few recent works listed in Table 3, DES were used as a modifier of typical stationary phases, such as silica gel or organic polymer‐based particles. DES improved the separation in terms of selectivity, peak symmetry, and elution time. The results confirmed the potential of DES to achieve efficient separation of both hydrophilic and hydrophobic analytes. A detailed review based on DES as new functional materials applied in LC systems summarized the previous works including silica, carbon‐based materials, metal‐organic frameworks, and monoliths [36].

**TABLE 3:** DES as a part of stationary phase.

Analyte	Stationary phase	Mobile phase	DES	Method	Time (min)	Ref
Nucleosides/bases	DES‐hydrogel at silica	ACN:water 85:15	NMA/LiTFSI‐DES	LC	6	[37]
Vitamins B		80:20			8	
Phenyl ketones		25:75			8	
Polycyclic aromatic hydrocarbons		35:65			12	
Phthalates		25:75			16	
Alkylbenzenes		25:75			8	
Aromatic acids (13)^a^	Hydrophobic DES pseudo‐stationary phase	HP‐γ‐CD, 0.3% DES	(‐)‐Mentol‐octanoic acid	EKC	˂30	[38]
Tioconazole, isoconazole, miconazole, econazole enantiomers	Hydrophobic DES pseudo‐stationary phase	CM‐β‐CD, 1%	Methyltrioctylammonium Cl:OctA	EKC	—	[39]
Cu, Hg	PTFE powder modified with DES coating	Deionized water and thiourea in hydrochloric acid	Thymol:1‐(o‐tolyl)thiourea	ICP‐OES	15	[40]
Pb, Cd, Hg, Cu	PTFE microcolumn with glass fibre as DES carrier	—	Thymol: thionalide	ICP‐OES	—	[41]
Thymidine, inosine, guanosine	Poly(DES) at silica	ACN:water 90:10	ChCl:EG 1:3 DAC:EPB 1:1	HPLC	6	[42]
Benzene derivatives (6)^a^		35:65			16	
Phthalate derivatives (6)^a^		40:60			12	
Ketone derivatives (5)^a^		35:65			12	
Polycyclic aromatic hydrocarbons (5)^a^		35:65			18	
Steroid hormones (5)^a^		35:65			12	
Bisphenols (4)^a^		35:65			12	

^a^The number of compounds separated.

Abbreviations: CM‐β‐CD, carboxymethyl‐β‐cyclodextrin; DAC, (2‐(acryloyloxy)ethyl)‐trimethylammonium chloride; EKC, electrokinetic chromatography; EPB, ethyl paraben; HP‐γ‐CD, hydroxypropyl‐γ‐cyclodextrin; ICP‐OES, inductively coupled plasma optical emission spectrometry; LiTFSI, bis(trifluoromethane) sulfonimide lithium; NMA, N‐methylolacrylamide; OctA, octanoic acid; PTFE, polytetrafluoroethylene.

Zhao et al. compared DES hydrogel pore surface‐polymerized at silica stationary phase with commercial C18 columns/XAmide. A mobile phase containing a higher percentage of ACN, and a longer separation time were required to achieve separations with the latter. The adjustable composition of the DES hydrogel in terms of polarity resulted in efficient separation of both hydrophobic and hydrophilic analytes in HILIC and RPLC modes indicating universality of these columns. The real‐world application was demonstrated with the separation of PAHs in hand creams [37].

Li et al. used hydrophobic DES as a pseudo‐stationary phase in capillary electrokinetic chromatography (EKC), while menthol and octanoic acid in combination with cyclodextrins of different types were developed for the separation of isomers of aromatic acids. The ratio of hydrogen bond donor and acceptor in the DES mixture was studied with respect to the ion mobilities of critical pairs. All of the 13 acids can be separated in less than 30 min [38].

Similar system was applied by Xu et al. for chiral separation of enantiomers of different antimycotics, while DES based on methyltrioctylammonium chloride with octanoic acid in combination with cyclodextrin again created a pseudo‐stationary phase in EKC system. The critical aspects to be optimized were ratio of both components, cyclodextrin type and concentrations. When the separation was compared with surface active substances, polyoxyethylene dodecyl ether (Brij‐35) and tween‐20, the separation using cyclodextrin alone was the shortest, the combination of cyclodextrin with DES prolonged it a little, but the surface active substances significantly increased the migration times [39].

**FIGURE 4:**
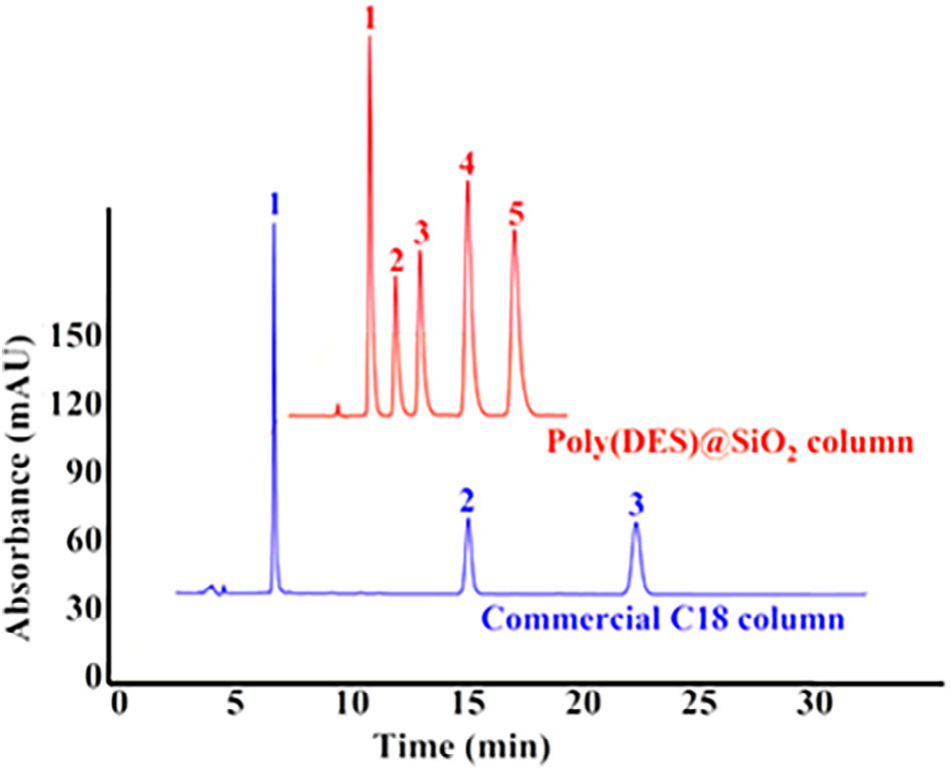
Separation of steroid hormones using the poly(DES)@SiO_2_ column and the commercial C18 column (Pronto SIL KromaPlus C18 column (150 × 4.6 mm, 5 µm). Conditions: mobile phase acetonitrile–water (35/65, v/v); temperature 25°C, flow rate 1.0 mL/min; detection 254 nm; Peaks: 1 prednisone; 2 hydrocortisone acetate; 3 norethindrone; 4 diethylstilbestrol; 5 medroxyprogesterone acetate. Reproduced with permission from ref. [42].

Liu et al. reported novel derivative of DES from 2‐(acryloyloxy) ethyl) trimethylammonium chloride and ethylparaben and polymerized it in the presence of ethylene dimethacrylate and silica particles to obtain mechanically stable deep eutectic supramolecular polymer functionalized stationary phase poly (DES)@SiO₂ [42]. Separations of a variety of hydrophilic and hydrophobic compounds demonstrated the universal applicability of this packing. Figure 4 compares the separation of steroid hormones using poly(DES)@SiO₂ and standard C18 column. While the former showed better selectivity and the separation required less mobile phase consumption due to shorter analysis time, the latter exhibited higher efficiency manifested by narrower peaks.

Shishov et al. developed DES‐based stationary phases and used them for the determination of elements such as copper and mercury in flow analysis [40, 41]. Their column was packed with polytetrafluoroethylene powder, which was pre‐soaked for 1 h in a DES prepared from thymol or menthol and 1‐(o‐tolyl) thiourea [40]. Alternatively, they used glass fibers as a support to fill the column and adsorbed DES prepared from thymol or menthol and terpenoid thionalide. They then compared their approach with other methods for the determination of metals in foods, such as wheat grits, rice, and powdered milk [41]. Their flow analysis system reduced human error, improved reproducibility, allowed full automation, and included an online sample preparation step. The use of natural, environmentally friendly solvents as the stationary phase and the automation of the process were in line with the closed‐loop management and the principles of green chemistry.

## 5 Summary and Outlook

DES features tunable physicochemical properties, low cost, biodegradability, and simple preparation. These characteristics have led to their use instead of conventional organic solvents. Their application range expands continuously. Despite a large number of reports published during the last 6 years covered by this review, the advancement remains inadequate, and the benefits of DES in chromatography have not been fully utilized. This can be changed by studying larger numbers of DES varying in hydrogen‐bond acceptor and hydrogen‐bond donor components in different molar ratios specifically designed for desired applications as mobile phases and their additives, and stationary phases. Their use in routine analysis of real samples also needs to be documented much more widely. One thing is certain. DES offers clear advantages over typical chromatographic methods, especially in terms of green/white chemistry principles. On the other hand, practicality in terms of easy optimization, clear evidence of improved separation efficiency compared to ACN as a mobile phase or C18 sorbent as a stationary phase, and coupling of modern separation trends with DES can help to broaden their applicability.

## Author Contribution

**Derya Demir**: writing–original Draft. **Joanna Antos**: writing–original draft. **Frantisek Svec**: writing–review and editing. **Hana Sklenářová**: writing–review and editing (equal), conceptualization, supervision, project administration, funding acquisition.

## Conflicts of Interest

The authors declare no conflicts of interest.

## Supporting information



Supporting Information

## Data Availability

Data sharing does not apply to this article as no new data were created or analyzed in this study.
