# Supplementary material for: Recent Developments in Deep Eutectic Solvents Applications in Liquid Chromatography: 2019–2025
Source: J Sep Sci. 2025 May 7;48(5):e70160. doi: 10.1002/jssc.70160 (PMC12056473; doi:10.1002/jssc.70160)

Supplementary Material

Recent developments in deep eutectic solvents applications in liquid chromatography: 2019-2025

Derya Demir[1], Joanna Antos[1,2], František Švec[1], Hana Sklenářová[1]*

[1] Department of Analytical Chemistry, Faculty of Pharmacy in Hradec Králové, Charles University, Akademika Heyrovského 1203, 500 03 Hradec Králové, Czech Republic

[2] Department of Water Supply and Bioeconomy, Faculty of Environmental Engineering and Energy, Poznan University of Technology, Berdychowo 4, 60-965 Poznan, Poland

Table of contents

Supplementary tables corresponding to White Analytical Chemistry [23] evaluation of articles mentioned in Table 1

Table S1: Red principles

Table S2: Green principles

Table S3: Blue principles

Table S4: Summarized results of White Analytical Chemistry evaluation

Table S1: Red principles

RED PRINCIPLES (analytical performance)			R1: Scope of application	R2: LOD and LOQ		R3: Precision			R4: Accuracy	
	**Method number**	**Method name**	0-100	LOD	0-100	RSD% (repeatability)	RSD% (reproducibility)	0-100	Recovery (%)	0-100
	**1**	**[15]**	100	225 nm	100	2.11-4.09%		80	94.34-101.48%	100
	**2**	**[16]**	100	225 nm	100	0.42-6.79%		70	95.79-114.53%	100
	**3**	**[17]**	100	240 nm	100	0.42-0.64%		100	98.93-101.03%	100
	**4**	**[19]**	90	254 nm	100	1.28-5.34%	0.86-4.91%	75	87.2-110.6%	90
	**5**	**[20]**	90	240 nm	100	0.64-1.82%		90	91.20-105.4%	100
	**6**	**[21]**	50	254 nm	100		1.74-4.80%	80		0
	**7**	**[14]**	90	283 nm	100	2.27-4.32%		80	98.78-100.01%	100

Table S2: Green principles

GREEN PRINCIPLES (green chemistry)			G1: Toxicity of reagents (impact and biodegradation)		G2: Amount of reagents and waste		G3: Consumption of energy and other media	G4: Direct impacts (safety, use of animals and GMOs)	
	**Method number**	**Method name**	Total number of pictograms	0-100	Waste production	0-100	1-100	Occupational hazards	Safety of users (0-100)
	**1**	**[15]**	**6.3**	87.5	10	95	0	2	90
	**2**	**[16]**	**9.3**	81.40	12	94	0	3	85
	**3**	**[17]**	**6.3**	87.4	1.2	100	0	2	90
	**4**	**[19]**	**3.8**	92	20	90	0	1	95
	**5**	**[20]**	**5.0**	90	15	92.5	0	2	90
	**6**	**[21]**	**3.3**	93	50	75	0	1	95
	**7**	**[14]**	**7.7**	85	26	87	0	2	90

Table S3: Blue principles

BLUE PRINCIPLES (practical side)	Method number	Method name	B1: Cost-efficiency		B2: Time-efficiency		B3: Requirements		
			Total cost	0-100	Speed of analysis	0-100	Sample consumption	Sample consumption (0-100)	Other needs: advanced instruments, skills, facilities (0-100)
	1	[15]	5.883	95	10	95	20	80	0
	2	[16]	7.68	95	12	94	20	80	0
	3	[17]	0.41	100	1.2	100	20	80	0
	4	[19]	35.28	80	20	90	20	80	0
	5	[20]	14.11	90	15	92.5	20	80	0
	6	[21]	96.04	70	50	75	10	90	0
	7	[14]	13.61	90	26	87	3	100	0

Table S4: Summarized results of White Analytical Chemistry evaluation

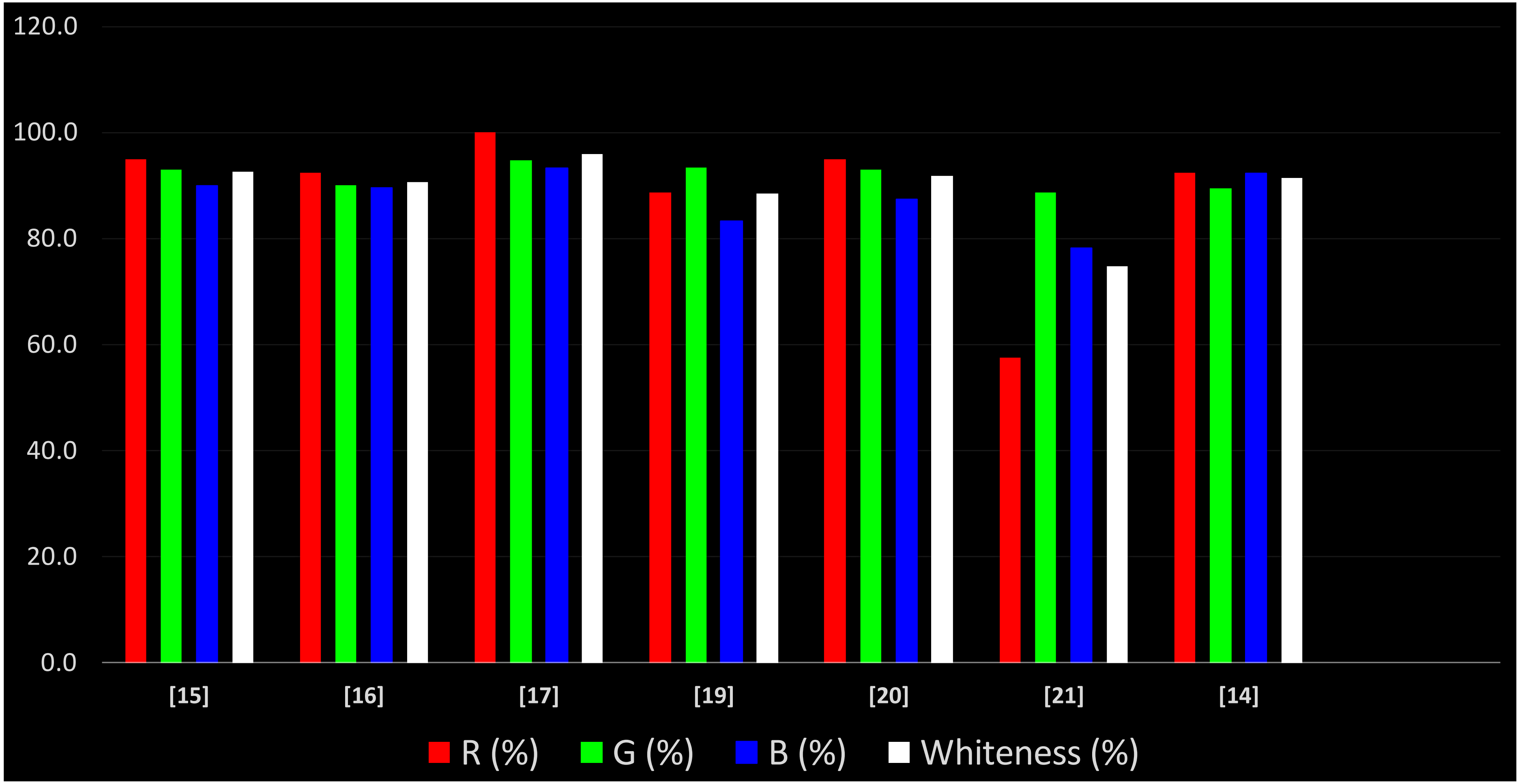

Supplement: Supplementary file 1 — Supporting Information [file JSSC-48-e70160-s001.pdf]
